# Tryptophan Levels as a Marker of Auxins and Nitric Oxide Signaling

**DOI:** 10.3390/plants11101304

**Published:** 2022-05-13

**Authors:** Pedro López-Gómez, Edward N. Smith, Pedro Bota, Alfonso Cornejo, Marina Urra, Javier Buezo, Jose F. Moran

**Affiliations:** 1 Institute for Multidisciplinary Research in Applied Biology (IMAB), Department of Sciences, Agrobiotechnology Building, Public University of Navarre (UPNA), Avda. de Pamplona 123, E-31192 Mutilva, Spain; pedro.lopez@unavarra.es (P.L.-G.); marina.urra@unavarra.es (M.U.); javier.buezo@unavarra.es (J.B.); 2 Department of Plant Sciences, University of Oxford, South Parks Road, Oxford OX1 3RB, UK; e.n.smith@rug.nl (E.N.S.); pedro.bota@plants.ox.ac.uk (P.B.); 3 Institute for Advanced Materials and Mathematics (INAMAT2), Department of Sciences, Public University of Navarre (UPNA), Ed. ‘Los Acebos’, Campus de Arrosadia, E-31006 Pamplona, Spain; alfonso.cornejo@unavarra.es

**Keywords:** tryptophan, indole-3-acetaldoxime, IAOx, nitric oxide, NO, stress, principal-component analysis

## Abstract

The aromatic amino acid tryptophan is the main precursor for indole-3-acetic acid (IAA), which involves various parallel routes in plants, with indole-3-acetaldoxime (IAOx) being one of the most common intermediates. Auxin signaling is well known to interact with free radical nitric oxide (NO) to perform a more complex effect, including the regulation of root organogenesis and nitrogen nutrition. To fathom the link between IAA and NO, we use a metabolomic approach to analyze the contents of low-molecular-mass molecules in cultured cells of *Arabidopsis thaliana* after the application of S-nitrosoglutathione (GSNO), an NO donor or IAOx. We separated the crude extracts of the plant cells through ion-exchange columns, and subsequent fractions were analyzed by gas chromatography-mass spectrometry (GC-MS), thus identifying 26 compounds. A principal component analysis (PCA) was performed on N-metabolism-related compounds, as classified by the Kyoto Encyclopedia of Genes and Genomes (KEGG). The differences observed between controls and treatments are mainly explained by the differences in Trp contents, which are much higher in controls. Thus, the Trp is a shared response in both auxin- and NO-mediated signaling, evidencing some common signaling mechanism to both GSNO and IAOx. The differences in the low-molecular-mass-identified compounds between GSNO- and IAOx-treated cells are mainly explained by their concentrations in benzenepropanoic acid, which is highly associated with IAA levels, and salicylic acid, which is related to glutathione. These results show that the contents in Trp can be a marker for the study of auxin and NO signaling.

## 1. Introduction

**Figure 1:**
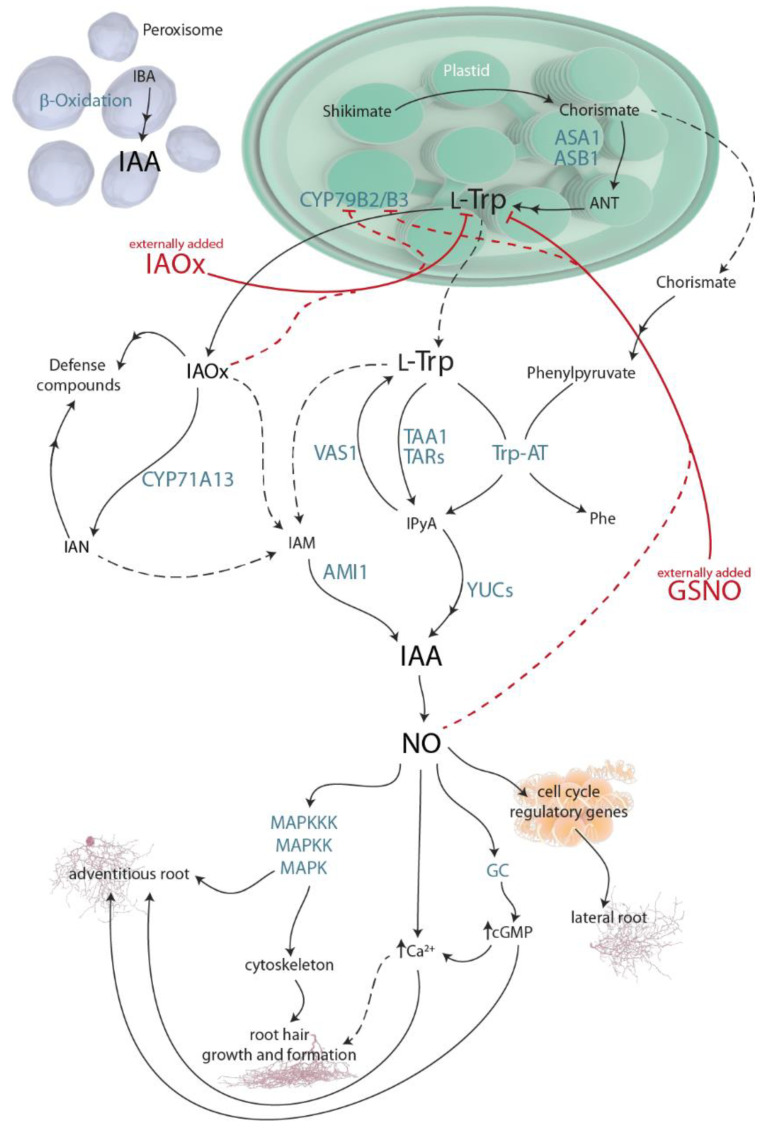
The main pathways for indole-3-acetic acid (IAA) metabolism in plants and the signaling networks involving auxin, NO, and cellular messengers. The main results of this article involving this pathway are represented by plain red lines. Possible implications derived from our results are indicated by dashed red lines. Plain arrow heads indicate inhibition/reduction. Plain black arrows represent described metabolic routes, while dashed black arrows describe not yet clear pathways. Organelles are not drawn to scale. AMIDASE-LIKE PROTEIN 1 (AMI1), anthranilate (ANTANTHRANILATE SYNTHASE α SUBUNIT 1), (ASA1), ANTHRANILATE SYNTHASE β SUBUNIT 1 (ASB1), cyclic GMP (cGMP), CYTOCHROME P450, family 79, sub-family B, polypeptides 2 and 3 (CYP79B2/B3), guanylate cyclase (GC), indole-3-acetamide(IAM), indole-3-acetonitrile (IAN), indole-3-acetaldoxime (IAOx), indole-3-butyric acid (IBA), indole-3-pyruvic acid (IPyA), mitogen activated protein kinase signaling (MAPK), phenylalanine (Phe), TRYPTOPHAN AMINOTRANSFERASE OF ARABIDOPSIS 1 (TAA1), TRYPTOPHAN AMINOTRANSFERASE-RELATED PROTEIN (TAR), TRYPTOPHAN AMINOTRANSFERASE (Trp-AT), REVERSALOFSAV3PHENOTYPE 1 (VAS1), and YUCCA flavin-containing monooxygenases (YUC).

The history of plant biology is inevitably intertwined with the discovery of auxin, followed by many years of research to understand its mode of action during growth and development. After decades of study on auxin metabolism, it has been established that the aromatic amino acid L-tryptophan (Trp) is the main precursor for indole-3-acetic acid (IAA) biosynthesis in plants. Trp is produced in the chloroplasts over the shikimate pathway, a route through which the majority of living organisms, excluding animals, produce aromatic amino acids [1]. Trp-dependent auxin biosynthesis is all but linear. It involves various parallel routes converging at the production of IAA; indole-3-acetaldoxime (IAOx), indole-3-acetamide (IAM), and indole-3-pyruvic acid (IPyA) are the most common intermediates. The prevailing pathway for IAA biosynthesis in plants is the IPyA route (Figure 1).

Parallel to the IPyA pathway, the Trp derivative IAOx is an intermediate in an IAA biosynthetic route that is yet to be fully understood. The conversion of Trp to IAOx is mediated by two isozymes from the CYTOCHROME P450 (CYP) monooxygenase family, CYP79B2 and CYP79B3 [2,3,4] (Figure 1). Even recently published reviews of IAA metabolism, such as [5], still refer to both the IAOx molecule and CYP79B2/3 genes as having been found exclusively in Brassica species [6], suggesting that this pathway is restricted to the Brassicaceae family. This misconception is deeply held in the IAA-research community, despite the evidence that shows that for the last 15 years, the CYPs conversion of Trp to IAOx has been widely distributed across the plant kingdom, i.e., maize, pine tree, or *Medicago truncatula* [7,8].

IAOx is a well-known precursor of indole glucosinolates (IGs) and camalexin, which serve as defense metabolites in plants [2,9,10]. Nevertheless, an increase in IAOx levels—through either genetic disruption of the IG pathway, overexpression of the genes associated with IAOx biosynthesis, or by feeding IAOx to plants in high-dose—results in elevated IAA contents and a distinct phenotype to that produced by IAA, called *superroot*, and entails the reduced growth of the main root and the formation of numerous lateral roots [4,8].

Plants have numerous ways of coping with biotic and abiotic stress; however, this extreme metabolic and developmental plasticity requires a complex regulatory network. One of the first plant responses to the environment involves reactive oxygen species (ROS) and reactive nitrogen species (RNS), which are key signaling molecules and regulate major physiological processes such as growth, development, resistance to biotic and abiotic environmental stimuli, and the progression of programmed cell death, through the activation of secondary messengers, the induction of gene transcription and changes in enzyme activity [11,12,13]. In addition, these signaling pathways are interconnected, forming a finely tuned regulatory system optimizing plant stress responses [11,14,15]. RNS is a term used to collectively refer to nitric oxide (NO) and the molecules derived from this radical, peroxynitrite (ONOO^−^)—the product of NO reaction with O_2_ *^−^—and other reactive nitrogen oxides such as nitrogen dioxide (NO_2_) and dinitrogen trioxide (N_2_O_3_). NO-derivatives of small molecules such as GSNO are also included in this group [13,16].

Although RNS play a key role in the regulation of multiple physiological processes, they are highly reactive molecules and can interact with almost any molecule in the cell. Therefore, besides their signaling function, RNS can be remarkably harmful to cells, mostly at elevated concentrations [17]. In view of this, RNS are necessary and tightly regulated participants of cell signal transduction, which may trigger adaptive responses. This is essential during the acclimation and survival of organisms under moderate stress. In contrast, through severe stress, their levels can increase enormously and may lead to serious damage, which includes cell death [17].

Even though NO has been extensively reported as a relevant signaling molecule in plants, neither its production nor its signal transduction mechanisms are fully elucidated [18]. This scene is in contrast with the achieved knowledge in mammals, where it is well established that most of the NO is synthesized by NOSs, sensed by guanylate cyclases (GCs) and signaled through multiple sophisticated pathways. Most of these processes and components remain unknown or mostly debated in plant research.

Many reports have described auxin and NO connection (reviewed in [19,20]) since 2002, when NO was shown to be required for adventitious root formation in cucumber [21]. Since then, it was found that NO provokes a downstream IAA response that promotes adventitious root development through pathways involving the GC-catalyzed synthesis of cGMP [22] and the activation of a protein kinase (MAPK) cascade [23] (Figure 1). Furthermore, high NO concentrations were detected with the fluorescent probe DAF-2DA in pericycle cells that originate lateral roots, indicating that NO is present during the early stages of lateral root development [24], where auxin is mainly implicated [20]. Moreover, treatment with the NO-donor SNP increased the lateral root number and, at the same time, decreased the primary root length in a dose-dependent manner [24], a correlated phenotype to that observed after IAA and IAOx treatments [8]. Furthermore, NO has been also reported to be implicated in hair differentiation and elongation in a similar manner to IAA [25].

The biosynthesis of NO remains under debate, but the way plants sense NO is even less known. NO perception in animals is performed through NO-inducible soluble GCs that synthesize the second messenger cGMP from guanosine triphosphate [26]. Although a flavin monooxygenase called NO-dependent guanylate cyclase 1, with higher affinity for NO than for molecular oxygen, was identified in *Arabidopsis* [27], it is not clear yet whether this enzyme produces enough cGMP to work as a true NO receptor [28]. It is also unknown whether enzymes involved in cGMP degradation and down-stream signaling, such as phosphodiesterases, are functional in plants [28], which makes the functionality of an NO–cGMP signaling pathway in plants even more uncertain [29]. In the absence of a GC receptor for NO, plants seem to sense NO mostly through chemical interactions with cofactor metals or with specific amino acid residues of proteins that undergo NO-triggered post-transcriptional modifications [30]. Alternatively, an NO-sensing mechanism involving the so-called Cys–Arg/N-end rule proteolytic pathway has been reported in *Arabidopsis* [31].

These unique challenges that NO research offers can confound the investigation, mainly due to the difficulty in detecting and quantifying biological NO production derived from the highly reactive nature of free NO. To overcome these obstacles, it has been recommended that researchers employ a combinatorial approach to monitoring NO concentrations, utilizing multiple methods with different detection principles. Most of them detect NO only after it has diffused outside of the cell or tissues, or they require cell extraction, such as the haemoglobin method [32], ozone-based chemiluminescence [33], electron spin resonance based on non-permeating spin traps [21], mass spectrometry [34], laser-photoacoustics [35], or amperometric methods with NO-specific electrodes [36]. Only the fluorophore diamino-fluorescein (DAF) has cell-permeable forms (DAF-2DA or DAF-FM) that are thought to indicate NO production inside cells [37].

Despite the fact that DAF has been preferentially applied in studies of plant NO-producing systems, it has many drawbacks. It has been proposed that DAF-2 does not react directly with the NO free radical but rather with nitrous anhydride (N_2_O_3_) [38]. N_2_O_3_ may be produced by the autoxidation of NO in air, and in aqueous solution it leads to NO_2_^−^ formation [38]. So, fluorescence intensity should not only rely on NO production but also on the rate of NO oxidation, which requires O_2_ and which should strongly depend on the NO concentration. Thus, it was suggested that DAF cannot be utilized under anoxia [39]. Moreover, the emission wavelength of DAF overlaps with that of PTIO—the most commonly utilized NO scavenger—masking its fluorescence and producing false negatives [40]. In the last decade, alternative probes have emerged, such as MNIP-Cu [41], but it still has a similar emission wavelength to that of DAF, thus sharing its problems with PTIO. Another technique is called the Griess assay, which is the method developed by Johann Peter Griess in the 19th century to detect nitrite by the formation of colored diazonium compounds in acidified solutions [42] and now is widely used in modern NO research. The practical sensitivity limit for this method is approximately 0.5–3 µM nitrite, depending on the matrix, which is becoming insufficient in recent studies. So far, ozone-based chemiluminescence is generally recognized as the most precise and sensitive technique available to measure NO and can be used to quantify it. However, it requires an expensive and specifically made apparatus, together with special training for its handling. In ozone-based chemiluminescence, NO reacts with ozone, producing excited-state NO_2_ (NO_2_ *), which, upon decay to the ground state, releases a photon that is detected by a photomultiplier [43].

The data obtained to this point with chemiluminescence, and the data published by numerous others based on DAF fluorescence, laser-photoacoustics, mass spectrometry, or haemoglobin, were partly incongruous [43]. This, together with the exceptional growth in plant NO research during the last thirty years, has opened many pathways in the quest for a significantly better understanding of how NO is produced and sensed and where and how NO wields regulatory functions. Here, we expose an analysis focus on the role of the Trp kay regulatory point in auxin biosynthesis and whether it can be a marker for auxin and NO signaling in plant.

## 2. Results

### 2.1. Ionic Phases Separation of Plant Cell Contents

The cells extracted from *Arabidopisis thaliana* and exposed to IAOx or GSNO were directly subjected to GC-MS assays. The column used was a low-polarity column suitable for a wide range of nonpolar and polar compounds. Furthermore, we generated methoimated-TBDMS derivatives since this facilitates the resolution of a wide range of low-molecular-weight metabolites and is used routinely for the analysis of amino acids, organic acids and related compounds in biological samples [44,45,46].

GC-MS assays from *A. thaliana* cells exposed to IAOx or GSNO soon revealed poorly defined chromatography profiles (Figure S1b). No convolution analysis could be performed directly on these samples to properly separate the peaks independently. Thus, several strategies were designed to facilitate it. A phase separation using formic acid and ether did not improve the results (data not shown). Finally, the utilization of ion exchange columns effectively separated the initial poor profile into three better defined profiles, each named corresponding to the phase from they were obtained—cationic, neutral, or anionic (Figure S1c–e). Once these three profiles were acquired, mass spectrometry analyses were performed. Indeed, peaks between 25- and 31-min retention times (RT) increased their definition. The improvement was even more severe in the range between 36- and 42-min RT, where most of the poorly defined peaks of this region were more defined and now distributed between the three obtained phases (Figure S1), facilitating its analysis.

### 2.2. Modification of Several Molecular Contents by IAOx and GSNO Exposure

A total of 26 molecules were detected by GC-MS and catalogued using MassHunter^®^ software and the Oxford Plant Sciences department GC-MS library of authentic standards (Table 1). The nature of these 26 molecules was quite heterogeneous. For example, 6 of them contained aromatic residues (imidazole, salicylic acid (SA), benzenepropanoic acid, uric acid, tryptophan, and 3-phenyl-1-butanone), while only one fatty acid was identified (Table 1). These 26 molecules participate in a broad number of metabolic processes, from amino acid biosynthesis (e.g., imidazole and urea) to the tricarboxylic acids cycle (e.g., fumarate, malate, and citrate) (Table 1). To ease the classification, the molecules were located within the plant molecular map using the Kyoto Encyclopaedia of Genes and Genomes (KEGG) (Table 1). Identified molecules obtained 211 appearances in 20 different KEGG maps (Table 1).

**Figure 2:**
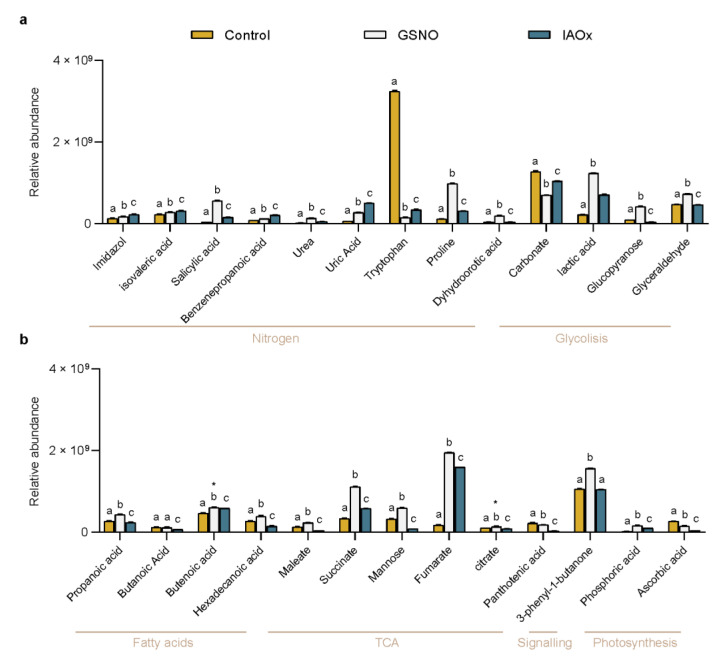
The relative abundance of (**a**) *Nitrogen-* and *Glycolysis*-tagged molecules and (**b**) *Fatty acids*, *TCA (Tricarboxylic Acid Cycle)*, *Signaling,* and *Photosynthesis*-tagged molecules identified in *A. thaliana* cells exposed for to 2 h to GSNO (light grey) or IAOx (blue) or no-treated cells (yellow). Molecules are sorted by their labeling. Letters denote the statistical differences (*p* < 0.001, * *p* < 0.05) between treatments within each molecule in a two-way ANOVA Tukey post-hoc analysis.

The relative abundance analysis showed how each treatment (Control, GSNO, or IAOx) differently modified these molecular contents (Figure 2). All of the studied molecules except for butanoic acid showed statistically significant differences between treatments (*p* < 0.05) (Figure 2). Especially, the decrease in the Trp relative abundance in both treatments (a 10-fold decrease in IAOx and a 25-fold in GSNO) compared to the control levels (Figure 2a) and, oppositely, the high increase in Fumarate in both treatments (a 9-fold increase in IAOx and an 11-fold in GSNO) are noteworthy (Figure 2b). Moreover, the high relative abundances of 3-phenyl-1-butanone and carbonate were obtained in the three studied treatments (Figure 2).

**Figure 3:**
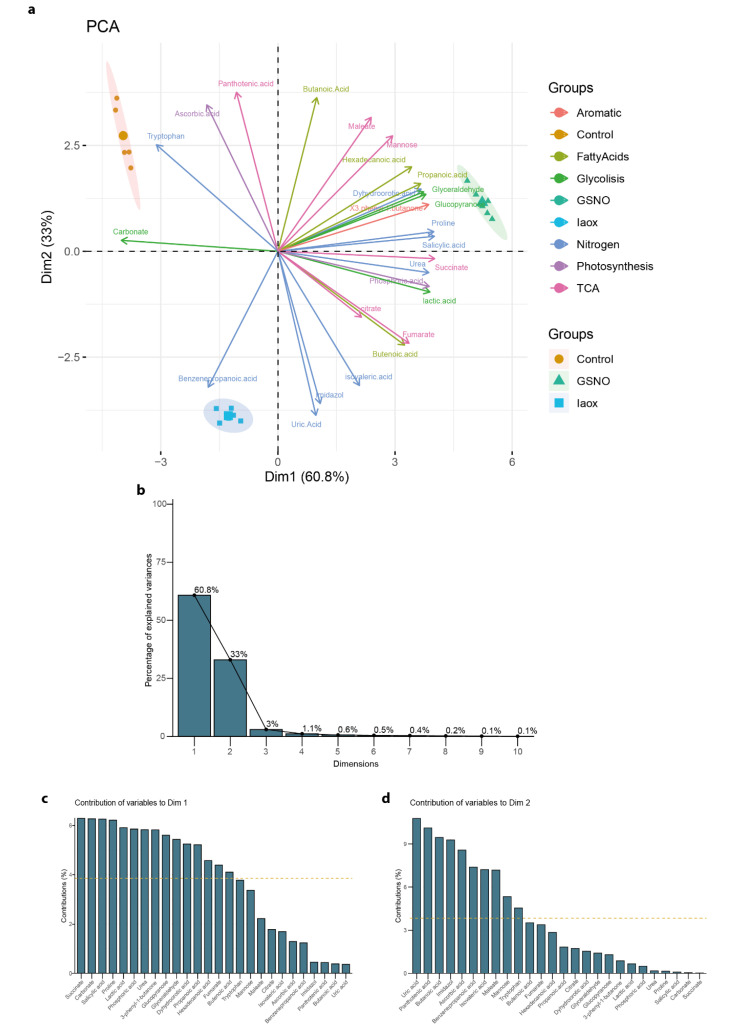
(**a**) The principal component analysis (PCA) of the 26 identified molecules in *A. thaliana* cells by GC-MS as variables (colored as indicated in legend). The area of each group of samples from the same treatment is depicted as follows: Control (yellow), GSNO (green), and IAOx (blue). The dot size corresponds to loading cos^2^. (**b**) The relative contribution (%) of each dimension to total variation. The relative contribution (%) of each variable to (**c**), Dim1 (PC1), and (**d**) Dim2 (PC2). The average contribution is delimited by a yellow dashed line.

However, the analysis of the levels of the compounds and the relations to the treatment were not obvious (Figure 2), even after results produced by an initial Principal Components Analysis (PCA) (Figure 3a). The general PCA clearly defined 3 groups, each corresponding to each treatment applied to the *A. thaliana* cells (Figure 3a). This PCA showed well defined dimensions (principal components (PC)), where the first principal component (PC1) explained 60.8% of the total variation and the second principal component (PC2) explained 33%, both covering up to 93.8% of the total variance (Figure 3b). PC1 mainly divided the Control from the GSNO treatment, while PC2 separated these two treatments from IAOx (Figure 3a). Nevertheless, this PCA did not clarify the main contributors to each dimension (Figure 3c,d). The main PC1 contributors were succinate, SA, L-proline, and carbonate, with each providing more than 6% of the total contribution (Figure 3c). On the other hand, PC2 is mainly formed by uric acid, pantothenic acid, and butanoic acid, contributing 10.8, 10.3, and 9.6%, respectively (Figure 3d).

With the aim of reducing the variables playing in the PCA, the 26 molecules were classified within the plant molecular map using the KEGG tool (Table 1). After, they were tagged accordingly with one of the arbitrary labels corresponding to the metabolism or process that they were related to: *Nitrogen, Glycolysis*, *Fatty Acids*, *TCA (tricarboxylic acids cycle)*, *Photosynthesis*, and *Signaling* (Table 1 and Figure 2). To focus on those candidates as markers of NO-related processes, once the 26 molecules were categorized only those molecules tagged with the *Nitrogen* label—imidazole, isovaleric acid, SA, benzenepropanoic acid, urea, uric acid, tryptophan, proline, and dyhydroorotic acid—were considered for further analyses. These molecules are related to amino acid biosynthesis (KEGG pathway ath00330 and ath01230), purine (KEGG pathway ath00230), and pyrimidine metabolisms (KEGG pathway ath00240).

**Figure 4:**
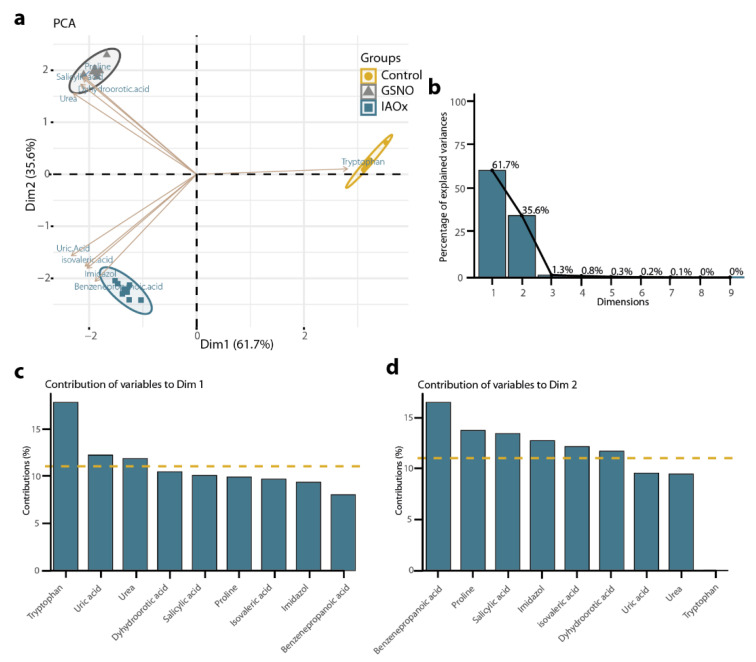
(**a**) The principal component analysis (PCA) using nitrogen-tagged molecules detected in *A. thaliana* cells by GC-MS as variables (brown). Area group samples of the same treatment, Control (yellow), GSNO (grey), and IAOx (blue). The dot size corresponds to loading cos^2^. (**b**) The relative contribution (%) of each dimension to total variation. The relative contribution (%) of each variable to (**c**) Dim1 (PC1) and (**d**) Dim2 (PC2). The average contribution is delimited by a yellow dashed line.

To outline metabolic traits in response to IAOx and GSNO exposition, a new PCA was performed with the mean values corresponding to the metabolic levels of these 9 molecules related to nitrogen metabolism from *A. thaliana* cells, which included a total of 21 observations (Figure 4a). The principal components vectors, the first principal component (PC1), and the second principal component (PC2) explained 97.3% of the total variation (Figure 4b). In this analysis, the PC1 accounted for 61.7% of the total variance, clearly separating the control group from the two studied treatments (Figure 4c). In contrast, PC2, which accounted for 35.6% of the variance, divided the three groups but significantly distanced the two treatments (Figure 4d). Thus, the molecules defining each PC can be considered as traits to the correspondent differences. In this case, PC1 was mostly defined by Trp and, to lesser extent, by urea and uric acid (Figure 4c), whereas PC2 was mainly defined by benzenepropanoic acid and mildly by proline and SA (Figure 4d).

In summary, this PCA analysis defined Trp, together with urea and uric acid, as a promising representative trait of molecular content differences between Control and IAOx/GSNO treatments, while benzenepropanoic acid, and proline and SA, was a potential marker to distinguish between GSNO and IAOx treatments (Figure 4). Ultimately, the groups of samples generated by both general and nitrogen-tagged PCA were coherent. Thus, the selection of molecules effectively improved and simplified the understanding of the effects that the treatments have provoked in terms of the differences in the relative abundances of the molecules identified.

## 3. Discussion

### 3.1. Ionic Phases Separation as an Accuracy Booster for GC-MS of Plant Material

The elevated levels of noise observed in the initial chromatography profiles (Figure S1) were probably the result of the high number and concentration of sugars in the crude extracts since their RT ranged between 22 and 44 min, as previously described [47]. However, the samples poured through an ion exchange column were successfully separated, increasing the resolution of the chromatography peaks and consequently those of mass spectrometry (Figure S1c–e). Thus, a total of 26 molecules of interest were effectively identified (Table 1 and Figure 2). Indeed, Dowex^®^ 50W X8(Thermo scientific. Waltham, MA, USA) resin has long been described as a successful tool for sugar separation of *Sedum* sp. plants [48]. It is noteworthy that the net surface charge of all molecules with ionizable groups is highly pH-dependent [49]. Therefore, the pH of the mobile phase should be selected according to the net charge of the molecule or molecules of interest.

### 3.2. Trp Mainly Defines the Observed Differences in the Molecular Contents Induced by NO

We categorized the 26 molecules according to their role in plant metabolism using KEGG, from which 9 molecules tagged as *Nitrogen* role were selected (Table 1 and Figure 2). The subsequent PCA analysis revealed two main groups: one defined by PC1, which separated the control from both the IAOx and the GSNO treatment, and the other group defined by PC2, which differentiated the IAOx from the GSNO treatment (Figure 4). The main characteristic that separated the GSNO and the IAOx treatment was the capacity of producing NO and the IAA-biosynthesis pathway relation, respectively. Since Trp is the main molecule by which PC1 was defined; we can now conclude that Trp is the principal marker—from the quantified molecules—for the effects of increased exposition of NO to *A. thaliana* cells. The role of Trp in IAA biosynthesis is well established, given that Trp is the main precursor of IAOx mediated by CYP79B2 and CYP79B3 [2]. The acute reduction in Trp contents after GSNO or IAOx application shows that either NO or IAOx reduce the Trp synthesis, or they increase Trp degradation. This emphasizes the signaling role that NO plays within this route. Indeed, this correlates with the rise on tryptophan decarboxylase, which converts Trp into tryptamine, provoked by an increase in NO in rice plants [50]. Furthermore, cytochrome P450 family is irreversibly inhibited by NO, so it is possible that the cytochrome P450 proteins CYP79B2/B3 were inhibited by NO too [2,51]. However, it is not demonstrated yet that the IAOx could release NO, but here it was demonstrated that IAOx and GSNO could share a regulatory role in *A. thaliana*. Moreover, oximes have been reported to be able to release NO in basic pH (Ph = 12) circumstances, where oximes are strongly ionized [52]. However, the oximes’ capacity to produce NO at pH = 12 was intimately linked to the group that accompanied the oxime group, where only those oximes linked to a pyridine ring were able to release some NO [52]. Nevertheless, these authors also suggest that in the observed chemistry in their experiments lie similarities with NO biosynthesis by NOS from L-arginine [52], converting IAOx into a promising but not yet confirmed NO source that might be inhibit CYP79B2/B3, acting as a product inhibitor. Since IAOx is quite a reactive molecule, plants tightly regulate its metabolic concentration [8]. Thus, the strict control of the concentration of its precursor Trp, with is indeed less harmful, could be vital for its homeostasis.

Two further molecules define this PC1: urea and uric acid. The former is part of the urea cycle and is directly related to both L-Arg and ammonia, and the latter is part of the purine degradation pathway, which ultimately leads to urea production [53]. The fact that NO is demonstrably related to these pathways [54,55,56,57,58] validates the results obtained by this unsupervised learning analysis.

PC2, which shows the differences in molecular contents that are not a direct consequence of NO, is mainly defined by benzenepropanoic acid (Figure 4d). This molecule, also named phenylpropanoic acid or hydrocinnamic acid, is a phenylpropanoid compound that is synthesized by plants from phenylalanine and tyrosine [59]. Specifically, benzenepropanoic acid derived from cinnamate in a reaction catalyzed by a type of oxidoreductase named 2-ENOATE REDUCTASE [60]. Phenylpropanoids contribute to a myriad of aspects in plant responses towards biotic or abiotic stimuli. They are, inter alia, mediators of plant resistance towards pests, as well as indicators of light and mineral stresses [61] and resource providers for reproduction [62]. Interestingly, IAOx-treated plant cells showed higher levels of benzenepropanoic acid than GSNO-exposed and control cells (Figure 2), which highlights the most likely relationship between phenylpropanoids and IAA-related molecules, as [63] emphasized, showing increased levels of phenylpropanoids in IAA-treated *Fagopyrum esculentum* plants. It is tempting to speculate that IAOx dependent-NO production somehow increases these phenylpropanoid contents within the plant but not the NO directly, in virtue of the low levels observed in GSNO-treated *A. thaliana* cells.

Another of the molecules that arose from the PCA analysis as one of the main definitors of PC2 was SA (Figure 4d). The biomolecule SA is a key regulator in mitochondria-mediated defense signaling and programmed cell death [64]. A loop between SA and ROS production in the defense response to stress was first reported almost three decades ago [65]. Later, it was demonstrated that ROS signals are involved in both downstream and upstream SA signaling in response to stress [66]. Remarkably, the evidence shows that SA not only plays a role as a pro-oxidant but also plays an antioxidant role together with glutathione (GSH) in the response to stress [66]. This correlates with its observed increased level in *A. thaliana* cells exposed to GSNO (Figure 2), compared to IAOx-exposed and control treatments. Therefore, these differences are probably the consequence of the very likely rise in both NO and GSH derived from the exposition.

Thus, the molecules that define PC1 and PC2 will be mainly responsible for the observed differences and, in the last term, these may be associated with the characteristics of these molecules. Furthermore, future studies on the effects of NO exposition and/or IAOx derivatives are needed. Hence, Trp and benzenepropanoic acid function will need to be revisited as markers of increased levels of NO in plants. However, the similarities in the reduction of Trp levels, both when GSNO—the source of NO—and IAOx were externally added to *A. thaliana* cell cultures, show that Trp is a key regulatory point of IAA biosynthesis. Likewise, Trp levels have been shown to be a useful marker for auxin and NO-associated signaling.

## 4. Materials and Methods

### 4.1. Plant Cell Culturing

The culture of plant cells was performed as described in the following method [67].

The cell line of *Arabidopsis thaliana* (L.) Heynh. (ecotype Landsberg erecta) was maintained in liquid MS medium supplemented with 166 mM glucose and subcultured every 7 days by transferring 10 mL of cell suspension into 90 mL of fresh MS medium containing glucose in a 250 mL conical flask. MS medium was prepared by dissolving 4.3 g Murashige and Skoog basal salt mixture salts (Sigma-Aldrich, St. Louis, MO, USA, M5524), 0.5 mg of naphthaleneacetic acid (50 μL from stock solution: 10 mg·mL^−1^ in ethanol), and 0.05 mg of kinetin (50 μL from stock solution: 1 mg·mL^−1^) in deionized water. Later, the pH was set to 5.8 using 1 M KOH and adjusted to a final volume of 1 L.

The flasks were placed on an orbital shaking platform rotating at 80–100 rpm, which was sufficient to allow for the continuous mixing of the cell suspension after addition. The flasks and their contents were equilibrated at the desired incubation temperature of 22 °C, for 15–30 min. After this time, 1 mL of the 7-day-old (dark-grown and light-grown separately) cell suspension culture was added to each flask. Immediately after the addition of the cell suspension culture, each flask was sealed with a rubber bung to avoid any contamination.

### 4.2. IAOx and GSNO Cell Exposition

From these cell cultures, 1 mL aliquots were obtained and firstly placed on 4 cm diameter paper discs. For their dispersion to be homogeneous over the entire surface of the disc, the disc paper was placed in a ceramic container attached to a 250 mL conical flask that continued to a vacuum pump. Thus, when the cells were placed on the filter, the pump was activated to homogeneously spread the cells over the surface of the disk. These discs containing A. thaliana cells were placed on Petri dish moisture with MS medium with 166 mM glucose and 0.5% agar and were grown for 5 days in the dark. After that period of time had elapsed, the same discs were transferred to a new Petri dish for 2 h, this time containing MS medium with 166 mM glucose and 0.5% agar with either 200 μM GSNO (Sigma-Aldrich, San Luis, MO, USA) or 200 μM IAOx (from a 200 mM stock in DMSO). After the treatment, the cells were collected in aliquots of 200 mg into 2 mL tubes containing 200 mL of ethanol and immediately preserved in liquid nitrogen. A proportional amount of DMSO was used for the control samples. IAOx was synthesized as described in [8].

### 4.3. Separation of Sample Phases by Ion Exchange Columns

The columns were prepared by placing approximately 10 glass beads (1.5–2 mm in diameter) in 10 mL tips sustained with a scaffold that maintained the columns in vertical position, and 500 µL of either the anionic (strongly basic Dowex 1X8; 200–400 mesh, (Thermo scientific, Waltham, MA, USA)) or cationic (strongly acidic Dowex^®^ 50W X8; 200–400 mesh, (Thermo scientific, Waltham, MA, USA)) exchange resin was added to several tips, allowing the solutions to run off. To charge the anionic resins, they were anionic washed three times with an excess of 1 M sodium acetate and then with 0.1 M acetic acid until the poured liquids were acidic. Parallelly, the cationic resins were washed three times with an excess of 2 M HCl and then with distilled water until the pH was the same as that of the water. Once activated, each cationic exchange resin tip was placed on top of an anionic exchange resin tip and a falcon tube at the bottom to collect the neutral fraction. Once the pH of the sample was fixed to 3–5, exactly 100 mg of cell extract was poured to the top and allowed to run in. Later, the columns were washed through with 3 × 833 µL of dH_2_O and the neutral fraction in the tube collected. Subsequently, the pipette tips were transferred to separate tubes, and 2 × 1 mL of 1 M NH_4_OH was added to the cationic column to elute the basic fraction. On the other hand, 2 × 1 mL of 2 M formic acid was added to the anionic column to elute the acidic fraction. Finally, collected fractions were preserved in liquid nitrogen.

### 4.4. Molecules Preparation for GC-MS and Derivatization

Sample preparation and derivatization procedures were based on [68].

Frozen samples were thawed at RT and resuspended by vortexing for 10–20 s. Subsequently, they were shaken for 10 min at 70 °C in a thermomixer at 950 rpm. Then, the samples were centrifuged for 10 min at 11,000 g, and the supernatant were transferred to a Schott GL14 glass vial. Next, 750 µL of chloroform and 1500 µL dH_2_O were added and vortexed for 10 s. Afterwards, the samples were centrifuged for 15 min at 2200 g. Two phases were obtained and collected, of which 150 µL was transferred from the upper phase (polar phase) into a fresh 1.5 mL tube. Samples were dried in a vacuum concentrator without heating for 24 h, collected, and frozen at −80 °C.

Frozen samples were placed in a vacuum concentrator for 30 min before derivatization. Additionally, one derivatization reaction using an empty reaction tube was prepared as a control. In a fume hood, 40 µL of methoxyamine hydrochloride (Sigma, cat. no. 593-56-6) at 20 mg·mL^−1^ in pure pyridine (Merck, Darmstadt, Germany, cat. no. 110-86-1) at 20–25 °C was added to each sample and was shaken for 2 h at 37 °C. Later, 70 µL of MtBSTFA + 1% TBDMS was added to the sample aliquots in a fume hood and shaken for 30 min at 37 °C. Finally, the samples were transferred into glass vials suitable for GC-MS analysis.

### 4.5. GC-MS Parameters

One microliter of each sample obtained previously was injected at 230 °C in splitless mode with a helium flow as a carrier gas set to 1 mL·min^−1^ by using the autosampler Agilent 7693 (Agilent Technologies, Santa Clara, CA, USA). The flow rate was kept constant with electronic pressure control enabled. The chromatography was performed in a Intuvo 9000 GC (Agilent Technologies, Santa Clara, CA, USA) with two low-polarity capillary columns of 15 m × 250 μm × 0.25 μm mesh (Agilent part number 122-5512UI-INT) coupled with a mid-column flow chip. The temperature program was isothermal for 2 min at 80 °C, followed by a 15 °C per min ramp to 330 °C. This temperature was held for 6 min. Cooling was as rapid as instrument specifications allowed. The transfer line was set to 250 °C. For the mass spectrometry, a 5977B MSD (Agilent Technologies, Santa Clara, CA, USA) detector was used. The ion source was set to 250 °C. The recorded mass range was *m*/*z* 70 to *m*/*z* 600, at 20 scans per s. The remaining monitored chromatography time proceeded with a 170-s solvent delay with filaments turned off. The manual mass defect was set to 0, the filament bias was 70 V, and the detector voltage was set between 1700 and 1850 V.

### 4.6. Data Analysis

GC-MS retention times and *m*/*z* were recorded with MassHunter software from Agilent Technologies (Santa Clara, CA, USA). Graphs were designed using GraphPad Prism 9.0.2., except for those related to PCA analysis, which were obtained by the *ggplot2* package for R in an RStudio environment. The following libraries were used for PCA analysis: *rstatix*, *factoextra*, *ggforce*, and *pca3d*. The number of biological samples was *n* = 7 for each treatment. The statistical analysis of the differences in molecular concentrations was conducted using GraphPad Prism version 8.0.1 for Windows (GraphPad Software, San Diego, CA USA). The differences (*p* value ≤ 0.001) among treatments in the concentration of each molecule were evaluated by two-way ANOVA with Tukey’s multiple comparisons post hoc test.

## 5. Conclusions

Trp contents in *A. thaliana* cultured cells significantly and markedly decreased when the NO donor GSNO or the IAA precursor IAOx were applied.

The concentration of Trp arose as an important marker of auxin and NO signaling.

The differences observed between the two treatments—GSNO and IAOx—are mainly due to the amount of benzenepropanoic acid, a member of phenylpropanoids family, which is highly associated with IAA levels.

## Figures and Tables

**Table 1:** The list of molecules detected and identified by GC-MS in *A. thaliana* cells. All of the molecules that were detected were categorized by their role in plant metabolic pathways following KEGG annotations and the author’s criteria.

Kegg Entry	Name	Structure	Formula	Related Pathways	Pathways KEGG Maps	Tag
C01589	Imidazole	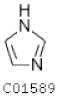	C_3_H_4_N_2_	Histidine and purine synthesis	map00340; map00230	Nitrogen
C08262	Isovaleric acid	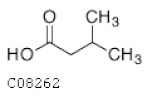	C_5_H_10_O_2_	Biosynthesis of alkaloids derived from histidine and purine	map01065; map01110; map04974	Nitrogen
C00805	Salicylic acid	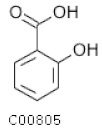	C_7_H_6_O_3_	Phenylalanine metabolism	map00360; map00621; map00624; map00626; map01053; map01061; map01070; map01100; map01110; map01120; map01220; map04075; map04976	Nitrogen
C05629	Benzenepropanoic acid	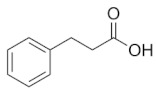	C_9_H_10_O_2_	Phenylalanine metabolism	map00360; map01100; map01120; map01220	Nitrogen
C00086	Urea	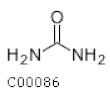	CH_4_N_2_O	Arginine and proline biosynthesis Purine metabolism Pyrimidine metabolism	map00220; map00230; map00240; map00330; map00780; map00791; map01100; map01120; map02010; map05120	Nitrogen
C00366	Uric Acid	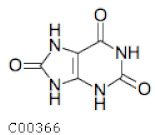	C_5_H_4_N_4_O_3_	Purine metabolism	map00230; map01100; map01120; map04976	Nitrogen
C00078	Tryptophan	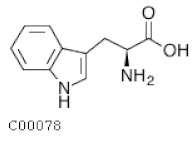	C_11_H_12_N_2_O_2_	Glycine, serine, and threonine metabolism Tryptophan metabolism	map00260; map00380; map00400; map00404; map00901; map00966; map00970; map00998; map01060; map01061; map01063; map01070; map01100; map01110; map01210; map01230; map01240; map04361; map04726; map04974; map04978; map05143; map05230	Nitrogen
C00148	Proline	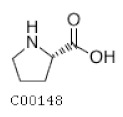	C_5_H_9_NO_2_	Arginine and proline metabolism	map00330; map00332; map00333; map00401; map00404; map00970; map01100; map01110; map01230; map02010; map04974; map04978	Nitrogen
C00337	Dyhydroorotic acid	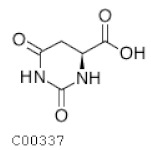	C_5_H_6_N_2_O_4_	Pyrimidine metabolism	map00240; map01100; map01240	Nitrogen
-	Carbonate	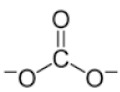	${\mathrm{CO}}_{3}^{2-}$	-	-	Glycolisis
C00186	Lactic acid	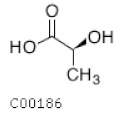	C_3_H_6_O_3_	Glycolisis and gluconeoge-nesis Fructose and mannose metabolism	map00010; map00051; map00620; map00640; map00643; map01100; map01110; map01120; map04024; map04066; map04922; map05230	Glycolisis
C00031	Glucopyranose	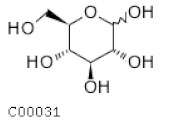	C_6_H_12_O_6_	Glycolisis and gluconeoge-nesis Pentose phosphate pathway	map00010; map00030; map00052; map00500; map00520; map00521; map00524; map00901; map01100; map01110; map02010; map02020	Glycolisis
C00577	Glyceraldehyde	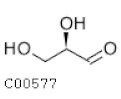	C_3_H_6_O_3_	Pentose phosphate pathway Fructose and mannose metabolism	map00030; map00051; map00052; map00561; map01100; map01120; map01200	Glycolisis
C00163	Propanoic acid	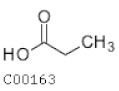	C_3_H_6_O_2_	Propanoate metabolism Ethylbenzene degradation	map00640; map00642; map00760; map01100; map01120; map01220; map04973; map04974	Fatty Acids
C00246	Butanoic Acid	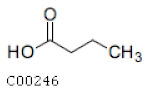	C_4_H_8_O_2_	Butanoate metabolism Carbohydrate digestion and absorption	map00650; map01100; map04973; map04974	Fatty Acids
C01771	Butenoic acid	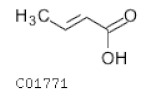	C_4_H_6_O_2_	Carbohydrate digestion and absorption	-	Fatty Acids
C00249	Hexadecanoic acid	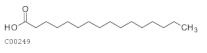	C_16_H_32_O_2_	Fatty acid biosynthesis, elongation, and degradation	map00061; map00062; map00071; map00073; map01040; map01060; map01100; map01212	Fatty Acids
C01384	Maleate	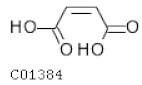	C_4_H_4_O_4_	Citrate cycle	map00350; map00650; map00760; map01100	TCA
C00042	Succinate	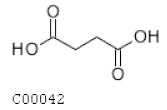	C_4_H_6_O_4_	Citrate cycle	map00020; map00190; map00250; map00310; map00350; map00360; map00361; map00620; map00630; map00640; map00650; map00720; map00760; map00920; map01060; map01061; map01062; map01063; map01064; map01065; map01066; map01070; map01100; map01110	TCA
C00159	Mannose	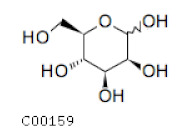	C_6_H_12_O_6_	Fructose and mannose metabolism Galactose metabolism	map00051; map00052; map00520; map01100; map02010	TCA
C00122	Fumarate	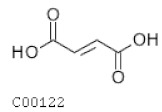	C_4_H_4_O_4_	Citrate cycle	map00020; map00190; map00220; map00250; map00350; map00360; map00620; map00643; map00650; map00760; map01060; map01061; map01062; map01063; map01064; map01065; map01066; map01070; map01100	TCA
C00158	Citrate	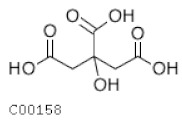	C_6_H_8_O_7_	Citrate cycle	map00020; map00250; map00630; map00997; map01060; map01061; map01062; map01063; map01064; map01065; map01066; map01070; map01100; map01110; map01120; map01200; map01210; map01230; map01240	TCA
C00864	Panthotenic acid	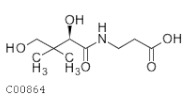	C_9_H_17_NO_5_	β-Alanine metabolism Pantothenate and CoA biosynthesis	map00410; map00770; map01100; map01110; map01240; map04977	TCA
-	3-phenyl-1-butanone	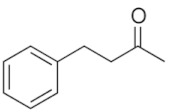	C_10_H_12_O	Attractant compound in flowers	-	Signaling
C00009	Phosphoric acid	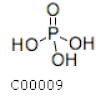	H_3_PO_4_	Photosynthesis	map00190; map00195; map02010; map04928	Photosynthesis
C00072	Ascorbic acid	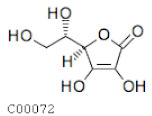	C_6_H_8_O_6_	Ascorbate and aldarate metabolism	map00053; map00480; map01100; map01110; map01240	Photosynthesis

## Data Availability

The data presented in this study are openly available in Figshare at https://doi.org/10.6084/m9.figshare.19761628.v1 and https://doi.org/10.6084/m9.figshare.19761649.v1.

## Supplementary Materials

The following supporting information can be downloaded at: https://www.mdpi.com/article/10.3390/plants11101304/s1. Figure S1: the chromatography profiles of an *A. thaliana* sample prior and after ionic phase separation. (a) The overlapping chromatography profiles of a TBDMs derivatized *A. thaliana* cell sample before ionic phase separation and the cationic, neutral, and anionic phases. (b) The chromatography profile of a TBDMs derivatized *A. thaliana* cell sample before ionic phase separation and of the (c) cationic, (d) neutral, and (e) anionic fractions after ionic phase separation. Video S1: a 3D view of the PCA analysis of the relative abundance of the 26 molecules of *A. thaliana* cells exposed to GSNO and IAOx.
