# Supplementary material for: Tryptophan Levels as a Marker of Auxins and Nitric Oxide Signaling
Source: Plants (Basel). 2022 May 13;11(10):1304. doi: 10.3390/plants11101304 (PMC9144324; doi:10.3390/plants11101304)
Supplement: Supplementary file 1 [file plants-11-01304-s001.zip › Figure S1.pdf]

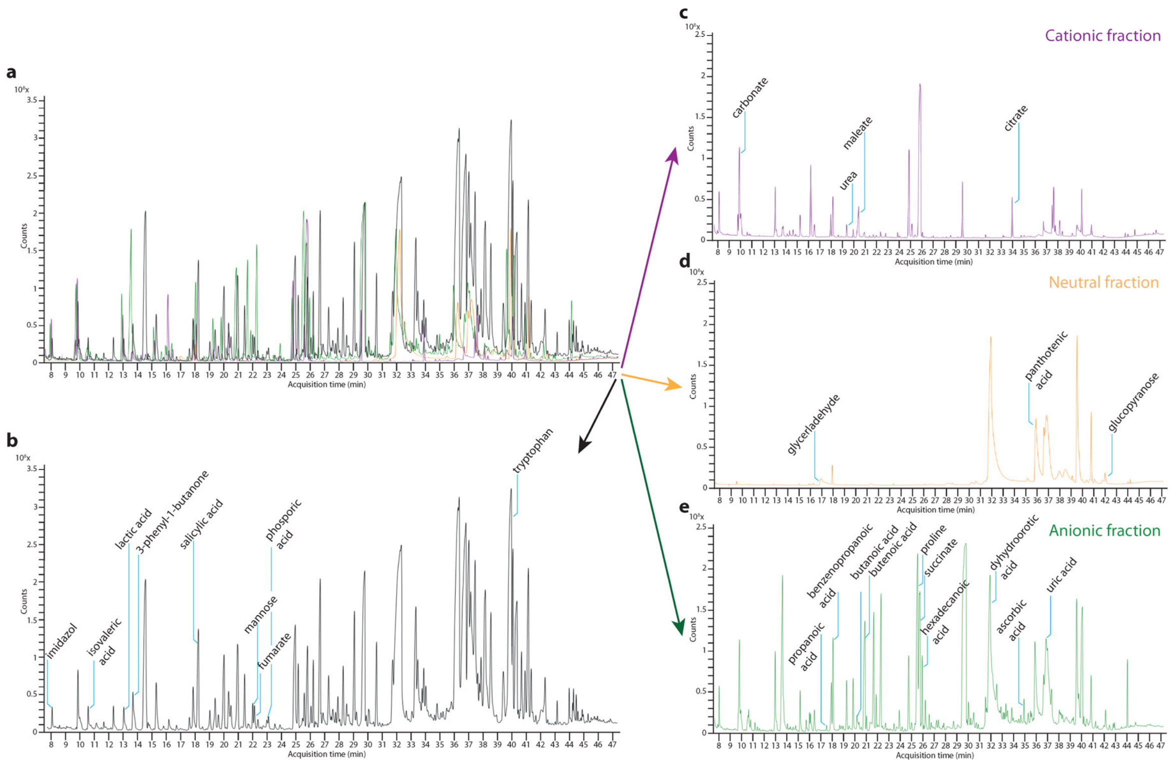

Figure S1. the chromatography profiles of an *A. thaliana* sample prior and after ionic phase separation. (a) The overlapping chromatography profiles of a TBDMs derivatized *A. thaliana* cell sample before ionic phase separation and the cationic, neutral, and anionic phases. (b) The chromatography profile of a TBDMs derivatized *A. thaliana* cell sample before ionic phase separation and of the (c) cationic, (d) neutral, and (e) anionic fractions after ionic phase separation.
